# Supplementary material for: Longitudinal change of cervical artificial disc motion following replacement
Source: PLoS One. 2020 Feb 25;15(2):e0228628. doi: 10.1371/journal.pone.0228628 (PMC7041810; doi:10.1371/journal.pone.0228628)
Supplement: Attachment — Submitted filename: Response to reviewers.docx [file pone.0228628.s001.docx]

**Response to reviewers**

Reviewer #1:

Thanks for your good comment. But we just followed up patients as scheduled time such as at 3 months, 6 months, 9 months, 12 months, 2 years, 3 years, 4 years, and 5 years postoperatively.

So we could not specify the time intervals at which ROM was assessed to further characterize the rate of decline. We are sorry about that.

Reviewer # 2:

Thanks for your good comment. We declare there are not any conflict of interest for this study.

All authors received no specific funding for this work
