# Supplementary material for: Longitudinal change of cervical artificial disc motion following replacement
Source: PLoS One. 2020 Feb 25;15(2):e0228628. doi: 10.1371/journal.pone.0228628 (PMC7041810; doi:10.1371/journal.pone.0228628)
Supplement: Attachment — Submitted filename: the response for reviewer #3.docx [file pone.0228628.s002.docx]

#1

Two observers in this study are spine surgeons. The first observer is Jung Hyeon Moon, M.D., first author, and the other observer is Won Heo, M.D., last co-author.

The each authors work as clinical professor in a different medical center at a different region.

They reached an agreement of in common about using tangent method for measurement.

The reliability of the radiologic measurements from two observers was confirmed with a Bland-Altman plot that is statistical method to confirm a correspondence.

We described these in materials and methods.

#2

All included patients had degenerative changes and correlating symptoms at the operated level.

For confirming a pure motion of artificial disc, we only included the patients who had healthy cervical facet joint (less than or equal to facet joint degeneration grade 1) with the careful screening of the preoperative CT. we described these in radiological assessment.

#3

We could not compare outcomes by cervical level within the cohort. Because the number of the operated levels were not even in this study.

For example, the number of patients who were operated at C5-6 level were 15, whereas the number of patient who was operated at c3-4 level was only 1.

We described these patient characteristics on Table 1.

#4

We tried to correct some grammatical errors. We highlighted changes in revised manuscript.
